# The Use of Clozapine in the Serious Mental Illness Patients Enrolled in an Assertive Community Treatment Program

**DOI:** 10.7759/cureus.15238

**Published:** 2021-05-25

**Authors:** Maria Ruiza Yee, Eduardo Espiridon, Adeolu O Oladunjoye, Udema Millsaps, Nailah Harvey, Anish H Vora

**Affiliations:** 1 Psychiatry, Drexel University College of Medicine, Philadelphia, USA; 2 Psychiatry, Philadelphia College of Osteopathic Medicine, Philadelphia, USA; 3 Psychiatry, Reading Hospital Tower Health, West Reading, USA; 4 Psychiatry, Tower Health Medical Group, West Reading, USA; 5 Medical Critical Care, Boston Children's Hospital, Boston, USA

**Keywords:** smi, act, clozapine, health care cost, community hospital

## Abstract

Introduction

Health care cost is projected to reach 20% of the nation’s gross national product (GNP) by 2016. 6.2% of this is from mental health. The National Institute of Mental Health (NIMH) estimates the prevalence of serious mental illness (SMI) at 13.1 million or 5.2% of American adults age 18 or over. Hence, mental health care cost for this patient population is significant.

Patients with SMI involved in an Assertive Community Treatment (ACT) program are individuals who experience the most intractable symptoms and the greatest level of dysfunction from their mental illness. These individuals typically are institutionalized in a long-term facility such as the state hospital.

Clozapine has shown superior efficacy over first- and most second-generation antipsychotics in both treating treatment-resistant and non-treatment-resistant schizophrenia which has been supported by several large trials. There is also evidence of its efficacy in suicidality, aggression and substance misuse. In fact, clozapine has been approved by the FDA for use in refractory schizophrenia and suicidality in schizoaffective disorder. Due to the risk of agranulocytosis, clozapine is underutilized. The purpose of this study is to conduct a retrospective cohort study through chart review to analyze whether the addition of clozapine to ACT treatment of SMI patients in a community hospital from 2008 to 2018 led to decreased frequency of hospitalizations and increased clinical stability.

Materials and methods

A retrospective study using electronic medical record (EMR) of patients ages 20 and above who were enrolled in the ACT program at a community hospital from December 1, 2008 to December 31, 2018. Variables were collected from the EMR and de-identified during data collation. Analysis was performed using SPSS software package.

Results

A total of 179 patients enrolled in the ACT program and their data was extracted from the EMR. Twenty-five (62.5%) of these patient enrollments were on clozapine. They were made up of 53.6% male, 81.9% White/Asian, 18.1% Black; 44.1% ages between 36 to 50 years old, 30.2% were aged 18 to 35 years old, and 25.7% greater than 50 years old. There was no difference in age, sex, race, ethnicity, and insurance type in ACT program between those using clozapine and those not on clozapine. There was a higher proportion of psychiatric hospitalizations among clozapine users compared with the non-clozapine user group (62.5% vs 41.5%, p = 0.019). However, the two groups did not differ from one another in terms of psychiatric emergency visits (p = 0.128) or frequency of ACT visits (p = 0.002).

Conclusion

Effective treatment that will reduce hospitalizations and the burden of chronic disability in patients with SMI would greatly reduce mental health care cost. Clozapine remains the gold standard in the treatment of refractory schizophrenia. But due to the risk of agranulocytosis, clozapine is underutilized. It was hoped that this study will support the use of clozapine in SMI patients. Disappointingly, the use of clozapine did not prevent relapses and hospitalizations in this patient population and patients on clozapine seemed to have increased hospitalizations, compared to those who were not on clozapine. Perhaps, a different outcome would have occurred if the focus was limited to the patients themselves who were on Clozapine and ascertain what the rate of hospitalization was before the start of clozapine vs after the use of clozapine.

## Introduction

Patients with serious mental illness (SMI) pose a significant mental health cost, not only due to the cost of acute care from inpatient psychiatric hospitalizations and frequent emergency room visits but also from indirect cost from loss of income from unemployment, the social services required to support these individuals in their chronic illness and various other indirect costs to support the chronic disability. 

Serious mental illness, as defined by the National Institute of Mental Health (NIMH) is a mental, behavioral or emotional disorder resulting in serious functional impairment which substantially interferes with or limits one or more major life activities [1]. SMI is made up of a smaller group of mental health illnesses and a more severe type of mental illness [2]. NIMH reports that approximately 13.1 million or 5.2% of American adults age 18 or over, as of 2019 have SMI [1], with the highest prevalence in young adults, ages 18 to 25 [1]. There is a higher risk in females as compared to males. Economically, SMI was estimated to cost the health care sector over 20% of the nations' gross national domestic product (GDP) in 2016 [3]. Mental health care constitutes 6.2% of health care costs in the United States. The mean reduction in earnings in persons with SMI is $16,306 [3]. Hence, it is important to prevent relapses and ensure clinical stability in this patient population.

Patients with SMI experience highly intractable symptoms and great levels of dysfunction from their mental illness [2]. Patients who suffer from SMI do not respond to standard outpatient psychiatric care and will need an alternative that is effective and efficient. Assertive Community Treatment (ACT) program is an effective [4-6], and cost-effective treatment [7-9] in this group of individuals, especially in those with extensive prior hospital utilization. ACT program not only results in a reduction of psychiatric hospitalization but also, more stable socioeconomic condition [2]. Patients with SMI involved in an ACT program are individuals who experience the most intractable symptoms and the greatest level of dysfunction from their mental illness. 

ACT program was started over 30 years ago by a group of mental health professionals at the Mendota Mental Health Institute in Wisconsin [5]. It started because of the high rate of recidivism in individuals with SMI who were discharged in stable conditions. They designed a service delivery model that is now recognized as the ACT program. A typical team membership includes 10 to 12 professionals from the different fields in mental health including fields of psychiatry, psychology, and other allied health care professionals who provided services needed by these patients [5]. Services are provided every time of the day and every day of the week [2]. Patients are taught life skills for real-life situations as well [2].

Prevention of relapse is very essential in SMI as psychopathology, social and occupational functioning worsen with repeated psychotic episodes [2]. Relapse can also lead to higher healthcare costs and hence an economic burden on a nation. Clozapine has shown superior efficacy than first- and most second-generation antipsychotics in both treating treatment-resistant and non-treatment-resistant schizophrenia which has been supported by several large trials [10-12]. There is also evidence of its efficacy in suicidality, aggression and substance misuse [12]. In fact, clozapine has been approved by the FDA for use in refractory schizophrenia and suicidality in schizoaffective disorder. Due to the risk of agranulocytosis, clozapine is underutilized [13]. The purpose of this study is to ascertain whether the addition of clozapine to ACT treatment of SMI patients led to a decrease in the frequency of hospitalizations and improvement in clinical stability.

## Materials and methods

This was a retrospective study conducted using data from the electronic medical record (EMR). The patient population studied included patients ages 20 and above who were enrolled at an outpatient ACT program at a community hospital from December 1, 2008 to December 31, 2018. Their data included diagnoses using the International Classification of Diseases (ICD-9) and ICD-10 codes. The ICD-10 diagnostic codes extracted were Manic episode F 30.x; Bipolar disorder F 31.x Schizophrenia F 20.x, and Schizoaffective disorder F 25.x. While the ICD-9 codes extracted were Schizophrenic disorders 295.x; Episodic mood disorders 296.x and Delusional disorders 297.x. Data on medications given during the study period to these patients were also extracted from the EMR. These included oral antipsychotic medications, both typical and atypical, and other medications administered during patient care in the ACT program.

A total of 189 patient enrollments in the ACT program were included in data collection. There were five patients who dropped out of the ACT program and five patients with incomplete/inappropriate data information which were excluded from the final analysis. Of the 179 patients remaining in the analysis, eight patients had two separate encounters in the ACT program while 163 patients had only one encounter in the ACT program. Patient hospitalizations were divided into two arms, i.e., those who received clozapine and those who did not receive clozapine.

To ascertain if clozapine was effective in reducing the number of hospitalizations, the number of hospitalizations was recorded after the start of clozapine versus the other arm where hospitalizations were recorded in those who were not on clozapine. Variables collected from the EMR were de-identified into an Excel spreadsheet for data collation and analysis was performed using SPSS software (IBM Corp., Armonk, NY) package.

Statistical analysis

In our statistical analysis, we described the prevalence of clozapine among SMI patients. Summary statistics were presented using proportions, mean and standard deviation. The mean number of hospitalizations, psychiatric emergency visits and number of planned ACT visits in the two groups (clozapine vs non-clozapine treatment) were compared.

Multivariate linear regression analysis was used to adjust for other independent co-variables. Kaplan Meier analysis was used to compare time from ACT enrollment to the first hospitalization. The level of significance was set at p < 0.05. All statistical analyses were performed using SPSS software package.

## Results

In our study, a total of 179 enrolments were analyzed. In 15 (37.5%) enrolments, clozapine was used. The enrolments were made up of 53.6% Male, 81.9% White or Asian, 18.1% Black, 44.1% ages between 36 and 50 years old. The overall mean age was 42.5 ± 11.8 years (Table 1). Table 1 describes the demographic and clinical characteristics of patients with SMI enrolled at the ACT program who were placed on clozapine. Insurance coverage varied with Medicaid insurance (56.5%) being the predominant insurance coverage, followed by Medicare insurance (36.2%), and private insurance or workman's compensation (7.3%). Most of the patients were unemployed or retired (95.9%). The most common diagnosis was schizoaffective disorder (44.0%), with schizophrenia (35.4%), bipolar disorder (16.0%) and major depressive disorder (4.6%) coming thereafter. Co-morbid substance abuse was significant with alcohol being the most commonly abused at 54.2%, followed by marijuana at 44.1% and cocaine at 29.6%. Most of the patients were enrolled in the ACT program for an average of one to five years (46.7%).

**Table 1:** Baseline and clinical characteristics of patients with serious mental illness (SMI) enrolled at the Assertive Community Treatment (ACT) program with the use of clozapine and without the use of clozapine. n = sample number; SE: Standard error; %: percentage.

Name	All SMI (n = 179)	Clozapine use (n = 40)	No clozapine use (n=139)	p-value
Mean age (±SE)	42.5±11.8	39.9±10.9	43.3±12.0	
Age, years				
18-35	54 (30.2%)	16 (40.0%)	38 (27.3%)	
36-50	79 (44.1%)	15 (37.5%)	64 (46.0%)	
>50	46 (25.7%)	9 (22.5%)	37 (26.6%)	0.306
Sex				
Female	83 (46.4%)	18 (45.0%)	65 (46.8%)	
Male	96 (53.6%)	22 (55.0%)	74 (78.3%)	0.844
Race				
White/Asian	145 (81.9%)	35 (89.7%)	110 (79.7%)	
Black	32 (18.1%)	4 (10.3%)	28 (20.3%)	0.151
Ethnicity				
Hispanic	44 (24.6%)	10 (25.0%)	34 (24.5%)	
Non-Hispanic	135 (75.4%)	30 (75.0%)	28 (75.5%)	0.944
Insurance				
Medicaid	100 (56.5%)	23 (57.5%)	77 (56.2%)	
Medicare	64 (36.2%)	15 (37.5%)	49 (35.8%)	
Private/ worker’s compensation	13 (7.3%)	2 (5.0%)	11 (8.0%)	0.810
Employment				
Unemployed/retired	162 (95.9%)	38 (97.4%)	124 (95.4%)	
Employed/self-employed	7 (4.1%)	1 (2.6%)	6 (4.6%)	1.000
Diagnosis				
Bipolar disorders	28 (16.0%)	3 (7.5%)	25 (18.5%)	
Major depressive disorder	8 (4.6%)	1 (2.5%)	7 (5.2%)	
Schizoaffective disorders	77 (44.0%)	19 (47.5%)	58 (43.0%)	
Schizophrenia	62 (35.4%)	17 (42.5%)	45 (33.3%)	0.295
Substance use disorder				
No	51 (28.5%)	15 (37.5%)	36 (25.9%)	
Yes	128 (71.5%)	25 (62.5%)	103 (74.1%)	0.152
Marijuana	79 (44.1%)	20 (50.0%)	59 (42.4%)	0.397
Cocaine	53 (29.6%)	13 (32.5%)	40 (28.8%)	0.649
Alcohol	97 (54.2%)	20 (50.0%)	77 (55.4%)	0.546
Smoking				
No	44 (24.6%)	13 (32.5%)	31 (22.3%)	
Yes	126 (70.4%)	27 (67.5%)	99 (71.2%)	0.138
Days in ACT program				
< 1 year	58 (42.3%)	7 (30.4%)	51 (44.7%)	
1-5 years	64 (46.7%)	10 (43.5%)	54 (47.4%)	
> 5 years	15 (10.9%)	6 (26.1%)	9 (7.9%)	0.034
Number of psychiatric hospitalizations				
0 No visit	94 (53.7%)	15 (37.5%)	79 (58.5%)	
≥ 1 visit	81 (46.3%)	25 (62.5%)	56 (41.5%)	0.019
Number of psychiatric emergency visits				
0 No visit	109 (64.1%)	21 (53.8%)	88 (67.2%)	
≥ 1 visit	61 (35.9%)	18 (46.2%)	43 (32.8%)	0.128
Planned ACT visits				
<100 visits	36 (20.6%)	5 (12.8%)	31 (22.8%)	
100-500 visits	82 (46.9%)	12 (30.8%)	70 (51.5%)	
>500 visits	57 (32.6%)	22 (56.4%)	35 (25.7%)	0.002

There was no difference in age, sex, race, ethnicity, and insurance type between those using clozapine and those not on clozapine. Most patients spent an average of 1-5 years in the ACT program (46.7%). When compared with the no clozapine group, a higher proportion of those in the clozapine group spent > 5 years in the ACT program (26.1% vs 7.9%, p = 0.034) and had at least one psychiatric hospitalization (62.5% vs 41.5%, p = 0.019). Of those who used clozapine, 46.2% (18/40) had at least one psychiatric emergency visit, whereas among those who did not use clozapine, 32.8% (43/139) had at least one psychiatric emergency visit. However, this was not statistically significant. The majority of planned ACT visits were between 100 and 500 visits during their program participation (46.9%). 

Association of psychiatric hospitalizations with clozapine use

Univariate analysis showed that clozapine use was associated with psychiatric hospitalizations (OR 2.30, 95% CI 1.25-4.24, p = 0.021). Males were less likely to have psychiatric hospitalizations (OR 0.47, CI 0.26-0.87, p = 0.015). Those with substance use disorders were less likely to have psychiatric hospitalizations compared to those who did not have substance use disorder (OR 0.46, 95% CI 0.24-0.90, p = 0.023) (Table 2).

**Table 2:** Association of psychiatric hospitalizations with clozapine use in serious mental illness (SMI) at the Assertive Community Treatment (ACT) program. *Univariate statistical significance; ^‡^accommodated univariate statistical significance of 0.1. Ref: Reference category used as analysis comparison.

Name	Univariate analysis (crude OR)	p-value	Multivariate analysis (adjusted OR)^‡^	p-value
Clozapine use	2.30 (1.25-4.24)	0.021*	2.41 (0.86 -6.77)	0.960
Age, years				
18-35	Ref			
36-50	0.96 (0.48-1.93)	0.909		
>50	0.93 (0.42-2.08)	0.866		
Sex				
Female	Ref			
Male	0.47 (0.26-0.87)	0.015*	0.26 (0.12-0.55)	<0.0001
Race				
White/Asian	Ref			
Black	0.81 (0.37-1.77)	0.596		
Ethnicity				
Hispanic	Ref			
Non-Hispanic	0.64 (0.32-1.28)	0.206		
Insurance				
Medicaid	Ref			
Medicare	0.99 (0.52-1.86)	0.964		
Private/worker’s compensation	0.99 (0.31-3.16)	0.987		
Employment				
Unemployed/retired	Ref			
Employed/self-employed	0.43 (0.08-2.29)	0.324		
Diagnosis				
Bipolar disorders	Ref			
Major depressive disorder	0.60 (0.12-3.01)	0.534		
Schizoaffective disorders	0.90 (0.38-2.14)	0.807		
Schizophrenia	0.85 (0.35-2.08)	0.719		
Substance use disorder				
No	Ref			
Yes	0.46 (0.24-0.90)	0.023*	0.89 (0.39-2.03	0.785
Marijuana	0.82 (0.45-1.50)	0.521		
Cocaine	0.64 (0.33-1.23)	0.179		
Alcohol	0.63 (0.35-1.15)	0.131		
Smoking				
No	Ref			
Yes	0.97 (0.48-1.94)	0.922		
Years in ACT program				
< 1 year	Ref			
1-5 years	1.90 (0.91-3.96)	0.088	1.80 (0.82-3.94)	0.143
> 5 years	2.50 (0.78-8.02)	0.124		
Planned ACT outpatient visits				
<100 visits	Ref			
100-500 visits	1.63 (0.71-3.74)	0.253		
>500 visits	1.81 (0.75-4.37)	0.185		

However, after adjusting for other covariates, there was no association between clozapine use and psychiatric hospitalization (OR 2.41, 95% CI 0.86 -6.77, p = 0.960). Sex remained an independent risk factor for psychiatric hospitalization with males being less likely to be hospitalized (OR 0.24, 95% CI 0.11-0.52, p < 0.0001) (Table 2).

**Figure 1:**
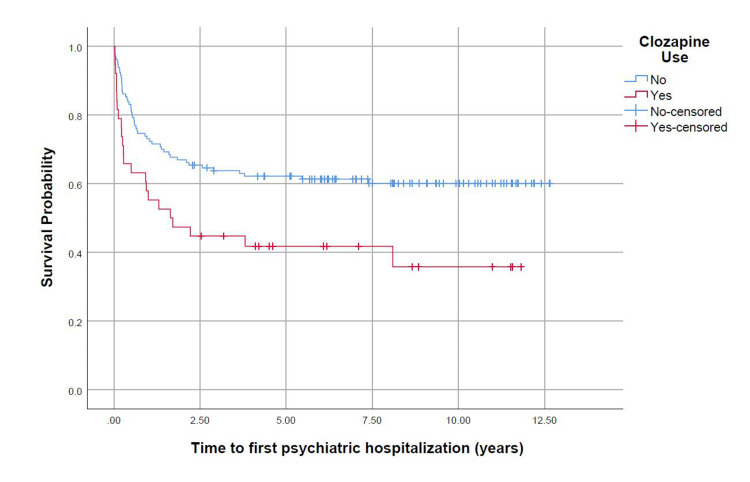
Plot comparing the probability of psychiatric hospitalization by clozapine use. Kaplan Meier curve analysis.

The mean time to first psychiatric hospitalization was 5.14 years for those on clozapine while it was 8.06 years for those not using clozapine (p = 0.008). At 1.638 years, 50% of those on clozapine and 33% of those not on clozapine had experienced a psychiatric hospitalization. The probability of psychiatric hospitalization at one and five years was higher in the clozapine group (45% and 59%, respectively) than in the no clozapine group (28% and 38%, respectively) (p = 0.008) (Figure 1).

## Discussion

Patients with SMI typically do not respond to standard outpatient psychiatric care [2]. Hence the introduction of ACT for SMI has made a remarkable difference in patients in the program. ACT has proven to be not only an effective [4-6] but also a cost-effective treatment [7-9] especially in those with extensive prior hospital use. The ACT program does not only lead to the reduction of psychiatric hospitalization but also, in more stable socio-economic conditions [2].

Clozapine has shown superior efficacy over first- and most second-generation antipsychotics in both treating treatment-resistant and non-treatment-resistant schizophrenia which has been supported by several large trials [10-12]. There is also evidence of its efficacy in suicidality, aggression and substance misuse [12]. In fact, clozapine has been approved by the FDA for use in refractory schizophrenia and suicidality in schizoaffective disorder. The patient outcome research team (PORT) initiated by the US Department of Health and Human Services recommends the use of clozapine in refractory schizophrenia after at least two adequate trials including at least one second-generation antipsychotic medication [13].

Due to the risk of agranulocytosis, clozapine is underutilized [14]. Other troublesome side effects such as excessive salivation, constipation, weight gain, seizure and rare significant risk of cardiomyopathy make it challenging for some patients and providers to use [15,16]. While clozapine remains underutilized, it has been shown to have benefits in reducing both positive and negative symptoms, and it is cost-effective in the long-term reduction in hospital admissions [17,18]. It has been reported, though, that many prescribers may, instead of transitioning to clozapine, prefer to use higher than PORT-recommended doses of other antipsychotics thereby delaying the use of clozapine in their patients [15,19]. Surprisingly, the results of our study do not substantiate the efficacy of clozapine in this patient population. This is despite the high level of adherence to clozapine prescribed while they were in the ACT program. If anything, it was associated with increased hospitalizations which is different from what is reported in previous studies.

It is worthwhile mentioning that the higher proportion of those on clozapine spent >5 years in the ACT program compared to those not on clozapine which was statistically significant p = 0.034 and those on clozapine had a higher proportion of patients with over 500 visits during their participation in the ACT program which was also statistically significant p = 0.002. Hence, this may indicate that this patient population is a more refractory subset of patients with SMI.

## Conclusions

Health care cost is one of the most daunting challenges of public policy in the United States. Hence, effective treatment that will reduce hospitalizations and the burden of chronic disability in patients with SMI would greatly reduce mental health care cost. Clozapine remains the gold standard in the treatment of refractory schizophrenia. But due to the risk of agranulocytosis, clozapine is underutilized. It was hoped that this study will support the use of clozapine in SMI patients. Disappointingly, the use of clozapine did not prevent relapses and hospitalizations in this patient population and in fact, patients on clozapine seemed to have increased hospitalizations, compared to those who were not on clozapine. It would seem that the use of clozapine was not contributory in maintaining clinical stability in this patient population. Perhaps, this is an indication that SMI patients in an ACT program constitute a very severe patient population and is not responsive to the use of clozapine. Perhaps, a different outcome would have occurred if the focus was limited to the patients themselves who were on Clozapine and ascertain what the rate of hospitalization was before the start of clozapine vs after the use of clozapine. 
